# Technology-based therapy-response and prognostic biomarkers in a prospective study of a de novo Parkinson’s disease cohort

**DOI:** 10.1038/s41531-021-00227-1

**Published:** 2021-09-17

**Authors:** Giulia Di Lazzaro, Mariachiara Ricci, Giovanni Saggio, Giovanni Costantini, Tommaso Schirinzi, Mohammad Alwardat, Luca Pietrosanti, Martina Patera, Simona Scalise, Franco Giannini, Antonio Pisani

**Affiliations:** 1 Dept. of Systems Medicine, University of Rome Tor Vergata, Rome, Italy; 2 Dept. of Clinical and Movement Neurosciences, Institute of Neurology, University College London, London, UK; 3 Department of Electronic Engineering, University of Rome Tor Vergata, Rome, Italy; 4 IRCCS Mondino Foundation, Pavia, Italy; 5 Dept. of Brain and Behavioral Sciences, University of Pavia, Pavia, Italy

**Keywords:** Parkinson's disease, Physical examination

## Abstract

Early noninvasive reliable biomarkers are among the major unmet needs in Parkinson’s disease (PD) to monitor therapy response and disease progression. Objective measures of motor performances could allow phenotyping of subtle, undetectable, early stage motor impairments of PD patients. This work aims at identifying prognostic biomarkers in newly diagnosed PD patients and quantifying therapy-response. Forty de novo PD patients underwent clinical and technology-based kinematic assessments performing motor tasks (MDS-UPDRS part III) to assess tremor, bradykinesia, gait, and postural stability (T0). A visit after 6 months (T1) and a clinical and kinematic assessment after 12 months (T2) where scheduled. A clinical follow-up was provided between 30 and 36 months after the diagnosis (T3). We performed an ANOVA for repeated measures to compare patients’ kinematic features at baseline and at T2 to assess therapy response. Pearson correlation test was run between baseline kinematic features and UPDRS III score variation between T0 and T3, to select candidate kinematic prognostic biomarkers. A multiple linear regression model was created to predict the long-term motor outcome using T0 kinematic measures. All motor tasks significantly improved after the dopamine replacement therapy. A significant correlation was found between UPDRS scores variation and some baseline bradykinesia (toe tapping amplitude decrement, *p* = 0.009) and gait features (velocity of arms and legs, sit-to-stand time, *p* = 0.007; *p* = 0.009; *p* = 0.01, respectively). A linear regression model including four baseline kinematic features could significantly predict the motor outcome (*p* = 0.000214). Technology-based objective measures represent possible early and reproducible therapy-response and prognostic biomarkers.

## Introduction

The increasing life expectancy has led in the past decades to a higher prevalence of age-related neurodegenerative diseases, including Parkinson’s disease (PD)^1^. In particular, PD results with median age-standardized annual incidence rates in high-income countries of 14 per 100,000 people in the total population, and 160 per 100,000 people aged 65 years or older^2^. Therefore, it has become crucial to have reliable and affordable diagnostic, prognostic, progression and therapy-response biomarkers, in order to support an early diagnosis and identification of more patients at higher risk of rapid motor progression, and to objectively evaluate patients’ response to therapy for customized therapeutic intervention since the early stages.

In this context, fluid biomarkers, either blood, or CSF samples, have been extensively investigated by studying molecules related to the pathophysiological mechanisms occurring in the disease, such as α-synuclein species, lysosomal enzyme activities, common Alzheimer’s disease biomarkers^3^. However, very few studies focused on the very early stages of the disease and on longitudinal data. Some works considered structural and functional neuroimaging-based progression biomarkers, but no definite data are available^4^. Indeed, the quantitative data from 123‐I Ioflupane dopamine transporter SPECT seem not to correlate to disease severity and progression, while preliminary data from magnetic resonance imaging morphometry suggest a correlation between atrophy and poor overall prognosis^5,6^.

To date, the assessment of the disease burden, of the progression and of the response to therapy still rely on patient-reported outcomes and clinical evaluation by means of validated rating scales. This approach can suffer from inter-rater and intra-rater variability and can be biased by the experience of the examiner. These are the reasons why an increasing interest emerged in technology-based assessment of motor function in PD patients. Several cross-sectional studies have focused on mild-moderate PD patients vs. healthy controls, to measure bradykinesia, gait abnormalities and postural instability using different technologies (RGB cameras, electromyography, inertial sensors, balance boards, and smartphones)^7–16^. Indeed, technology-based objective measures (TOMs) provide quantitative and reproducible data on motor performances, useful both for daily clinical practice and for scientific research^17^. In addition, TOMs can support the early identification of subclinical motor impairment or monitor response to therapy and patients’ activities over long periods^18–21^.

In previous works, we demonstrated objective detection of parkinsonian motor features in an initial phase of the disease by means of wearable inertial sensors on a cohort of de novo PD patients^20,22^. Here, we present results from TOMs performed in the same cohort of de novo PD patients prospectively followed up to nearly 30 months, in order to determine technology-based therapy response and to evidence prognostic biomarkers.

## Results

### Therapy response

Table 1 shows clinical and demographical data of 36 patients who completed the study at T0, T2, and T3. As expected, the MDS-UPDRS II and III scores decreased significantly after the initiation of the dopaminergic therapy. On the other hand, no significant differences were recorded in NMSS.

**Table 1:** Average and standard deviation of the clinical scores for patients at T0, T2, and T3.

	T0 (*n* = 36)	T2 (*n* = 36)	T3 (*n* = 36)	*p* value
Age (years)	62.7 ± 8.7			
Gender (M/F)	27/9			
Symptoms duration (years)	2 ± 2.04			
H&Y	1.7 ± 0.55	1.56 ± 0.5	1.54 ± 0.51	*p* = 0.32
MDS-UPDRS III	22.3 ± 9.14	17.3 ± 6.67	15.36 ± 6.75	*p* = 0.002
MDS-UPDRS II	6.9 ± 2.9	5.8 ± 2.9	5.3 ± 3.1	*p* = 0.007
NMSS	42.75 ± 35.49	40.25 ± 32.75	44.72 ± 30.31	*p* = 0.65
LEDD	0	273.86 ± 110.85	368.47 ± 132.2	*p* < 0.001

*M* male, *F* female, *H&Y* Hohen and Yahr stage, *MDS-UPDRS* Movement disorders society Unified Parkinson’s Disease Rating Scale, *NMSS* Non-Motor Symptoms Scale, *LEDD* Levodopa Equivalent Daily Dose.

**Fig. 1:**
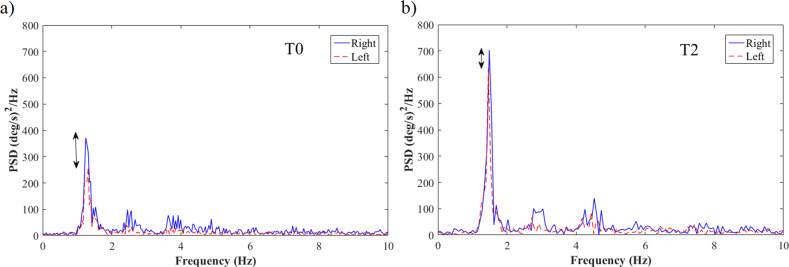
Evolution of RAHM amplitude after dopaminergic therapy initiation. The graphs show the power spectral density of the angular velocity measured by the sensor placed on right and left hand of a PD patient at T0 (**a**) and T2 (**b**). At the baseline (**a**), it is evident a reduced maximum value of the power spectral density and an asymmetry between right and left limb (measured by the feature Power and Asym_Power, respectively), which both improved with the therapy (**b**).

As reported in Table 2, several measured features from RAHM, LA, TT, PT, and TUG tasks significantly changed from T0 to T2. In particular, during all bradykinesia tasks (RAHM, LA, and TT) the movement amplitude increased (*Power*, Fig. 1). Differently, no improvements were evidenced in tremor related features. Several TUG features also improved, especially the ones related to the turning phase (*Turning Vel*, *Steps Turning*) and upper limb movement (*Arm Swing* and *Average Vel Arm*).

**Table 2:** Mean values and standard deviations of significantly improved RAHM, LA, TT, PT, and TUG features in PD patients from T0 to T2.

Feature (task)	[T0] Mean ± SD	[T2] Mean ± SD	*p* value	SRM
CV (RAHM)	11.39 ± 7.2	7.9 ± 3.18	0.002	−0.45
Amp_CV (RAHM)	21.25 ± 7.86	15.74 ± 5.39	<0.001	−0.59
Power (RAHM)	406.66 ± 158.45	528.14 ± 150.77	<0.001	0.77
Asym_Power (RAHM)	20.15 ± 10.37	13.29 ± 9.43	0.001	−0.61
Amp (LA)	29.07 ± 14.19	38.63 ± 24.12	0.012	0.39
Power (LA)	32.9 ± 17.35	44.61 ± 24.54	0.005	0.5
Amp (TT)	66.28 ± 22.96	78.14 ± 25.05	0.001	0.47
Power (TT)	50.14 ± 21.9	67.06 ± 26.9	<0.001	0.55
ROM ML (PT)	9.058 ± 5.133	13.72 ± 6.04	0.002	0.77
Number of Steps (PT)	2.156 ± 0.913	1.47 ± 0.8	0.017	−0.57
Turning Time (TUG)	2.073 ± 0.341	1.78 ± 0.24	<0.001	−0.72
Cv Stance (TUG)	0.08 ± 0.071	0.09 ± 0.06	0.026	0.18
Cv Swing (TUG)	0.107 ± 0.119	0.1 ± 0.05	0.033	0.03
Flex Arm (TUG)	24.901 ± 12.853	36.43 ± 18.15	0.033	0.5
Average_Vel_Arm (TUG)	16.774 ± 6.637	24.51 ± 8.27	0.007	0.54
Turning_Vel (TUG)	89.245 ± 15.466	102.77 ± 14.54	<0.001	0.7
Steps_Turning (TUG)	3.517 ± 0.871	2.93 ± 0.72	0.005	−0.54
Rms_ML_Sitwalk (TUG)	0.586 ± 0.18	0.76 ± 0.3	0.017	0.41
Flex_Trunk_Sitwalk (TUG)	37.98 ± 13.434	29.84 ± 18.03	0.048	−0.48

### Identification of technology-based prognostic biomarkers

To identify candidate technology-based prognostic biomarkers, we looked for correlations between baseline kinematic features and motor progression at T3, measured as the % variation of UPDRS III score between T0 and T3. Table 3 reports correlations between baseline motor features values and MDS-UPDRS score variation between T0 and T3. In particular, the average velocity of legs (*Average_Vel_Leg*) and arms (*Average_Vel_Arm*) during TUG test positively correlated with the percentage reduction of MDS-UPDRS III at T3 (respectively *r* = 0.451 *p* = 0.007 and *r* = 0.436, *p* = 0.009), while the *sit-to-stand time* showed an inverse correlation (*r* = −0.432, *p* = 0.01). From TT task, only the decrement in the amplitude (*Amp_decr*) was significantly inversely correlated with the improve in the clinical scale while the variability in the amplitude of the movement was only close to significance (respectively *r* = −0.433, *p* = 0.009; *r* = 0.296, *p* = 0.08). Features from RAHM and LA tasks did not reach statistical significance.

**Table 3:** Correlation coefficients for selected RAHM, LA, TT, and TUG features at T0 with MDS-UPDRS III score variation between baseline and last follow-up (T0 and T3).

Feature (task)	Correlation coefficients	*p* value
Amp_CV (RAHM)	−0.322	0.059
Asym_Power (RAHM)	−0.290	0.091
Amp_Decr (RAHM)	−0.291	0.090
CV (LA)	−0.327	0.055
Amp_CV (TT)	−0.296	0.084
Amp_Decr (TT)	−0.433	0.009
Average_Vel_Leg (TUG)	0.451	0.007
Average_Vel_Arm (TUG)	0.436	0.009
Sit-To-Stand Time (TUG)	−0.432	0.010

Then, linear regression was used to investigate variables predicting the motor progression at T3. Independent noncollinear variables showing an association at univariate analysis (*p* < 0.10) or significant correlation were then included in multivariate models, as appropriate. As shown in Table 4, the best multiple linear regression model to predict the clinical motor outcome included 4 features, since RAHM asymmetry (*Asym_Power*), LA coefficient of variability (*CV*), TT decrement (*Amp_Decr*), and TUG average velocity of legs (*Average_Vel_Leg*) reach excellent statistical significance (*p* = 0.000214; R square = 0.558875; adjusted R square = 0.480103). LEDD proved to be irrelevant (Standardized Beta = 0.006, *p* = 0.964).

**Table 4:** Multiple linear regression model to predict the motor outcome at T3, measured as the % variation in UPDRS III score between baseline and T3 (dependent variable).

Feature	B	Std. Error	S Beta	*p*	CI lower b	CI higher b
LEDD	0000017	0.000369	0.006386	0.964	−0.000738	0.000772
RAHM Asym_Power	−0.009001	0.004353	−0.281472	0.048015	−0.017918	−0.000084
LA CV	−0.023121	0.008178	−0.379282	0.008575	−0.039873	−0.006369
TT Amp_Decr	−0.024502	0.006125	−0.540302	0.000420	−0.037049	−0.011955
TUG Average_Vel_Leg	0.004229	0.001697	0.331834	0.018876	0.000753	0.007704

For each dependent variable beta (B), standardized beta (S Beta), standard error (std. Error), *p* values, and confidence interval (CI) are reported.

We also tested the power of the clinical assessment alone to predict the motor outcome. So, we built a multiple linear regression model including as independent variables the UPDRS III score at T0 and LEDD at T3 and as dependent variable the percentage of variation in UPDRS III between T3 and T0. This model was able to explain only the 13.5% of the variance (R square 0.187, adjusted R square 0.135, p 0.04).

## Discussion

The lack of reliable, reproducible, noninvasive, and affordable biomarkers for supporting the diagnostic process and monitoring the disease progression and the therapy response is one of the major unmet needs in PD^23,24^. However, the identification of early, reliable, measurable, objective, and noninvasive prognostic and therapy-response biomarkers is crucial for early stratification of patients and objective evaluation of the efficacy of a therapeutic intervention in PD. Indeed, validated rating scales, such as the MDS-UPDRS, or patient-reported questionnaires, such as the Hauser diary, are the most commonly utilized outcome measures for pharmacological and non-pharmacological intervention in PD^25–27^.

Here, we demonstrated that a dopaminergic replacement therapy ameliorates motor performances in a technology-based measurable and objective way, by means of wearable sensors.

Indeed, all tasks but tremor had at least one feature which significantly improved after therapy. The lack of efficacy on tremor is not surprising, and in agreement with the literature^28^. On the other hand, the task used for evaluating bradykinesia in upper limbs (RAHM) was the one with the most significant measurable improvement, consistent with clinical experience and previous findings^29–31^.

The identification of the most responsive features is extremely useful in evaluating the therapy efficacy, representing a valid and objective therapy-response biomarker, which could be helpful in evaluating outcomes in clinical trials^24,32^. In this regard, our findings suggest that the feature *Power*, a parameter related to the amplitude of the movement, emerges as a good candidate as therapy-response biomarker for all bradykinesia tasks. In fact, *Power* significantly improved after the dopaminergic therapy in all RAHM, LA, and TT tasks. Moreover, some TUG features related to the turning phase demonstrated a significant L-dopa responsiveness, in particular the turning time and velocity (SRM > 0.7). This is particularly interesting because gait disturbances are clinically detectable in later stages as compared to a kinematic gait analysis. A technology-based gait-related therapy-response biomarker could therefore have much higher sensitivity, mostly in earlier stages of the disease, allowing clinicians to customize the treatment even for subtle motor signs.

Another aim of this study was to identify early technology-based prognostic biomarkers. In particular, we followed up our cohort for at least 30 months and then we retrospectively analyzed patients’ baseline motor features in relation to their motor outcome defined as the MDS-UPDRS III variation at last follow-up. Interestingly, features from bradykinesia tasks (RAHM asymmetry, the variability of LA and the decrement at TT) and gait (velocity of legs) exhibited statistical significance in predicting the motor outcome at 30 months, whereas the LEDD did not have any influence, as demonstrated by the regression model. Accordingly, three features from TUG test, namely the speed of the upper and lower limbs while walking and the time needed to stand up from the sit position, and the TT decrement also significantly correlated to the clinical scores change at last follow-up. Of note, early overt gait abnormalities or signs in the lower limbs are typically considered red flags for alterative diagnosis other than idiopathic PD, mainly atypical parkinsonism^33^. This suggests that subclinical gait and lower limb motor abnormalities detectable with wearable sensors in the early phases could identify patients with less favorable prognosis and be therefore considered early prognostic biomarkers.

A note of caution should be added in our study, because of both the limited sample size and follow-up duration, which does not allow drawing conclusions to exactly determine a patient prognosis. Nonetheless, some features seem to be reliable prognostic biomarkers, showing a greater ability to predict the motor outcome than the baseline MDS-UPDRS III score alone. Once replicated in larger cohorts, these findings might allow early patients stratification, discriminating those with poorer or better prognosis. This would, in turn, help clinicians to refer more fragile patients to higher intensity cares involving pharmacological and non-pharmacological interventions, even in earlier disease stages.

Our findings come from an experimental laboratory setting, with the use of multiple sensors and evaluation of multiple tasks. Thus, on one hand, it might appear time-consuming for the clinical practice; nonetheless, on the other, the fact that most of the candidate features as prognostic biomarkers belonged to TUG test is extremely encouraging for clinical purposes. Indeed, lately, gait has been recognized as the outcome of complex integration of cortical and subcortical pathways, and subtle alterations of this feature are increasingly recognized as risk factors for several neurological diseases^34,35^. Moreover, a recent retrospective study on a large cohort of subjects not affected by neurological diseases demonstrated an association between longer TUG time and a later diagnosis of PD^36^. Therefore, a comprehensive and objective technology-based characterization of TUG test performance seems very promising to identify patients with worse motor outcome, even in earlier phases of the disease when motor impairment is very mild. In the hypothesis of integrating the technology-based analysis in clinical practice, the identification of few significant tasks will allow to optimize the technology-based evaluation.

In brief, a multimodality characterization of PD patients in the very early stages, including neuroimaging data, CSF and/or blood biomarkers, clinical assessment, demographics, and an objective technology-based characterization of motor performance is a promising approach to stratify patients with different progression rates, as also previously discussed^37^. However, in order to render this approach feasible in the clinical practice, further work is required to make these measures as affordable and quick as possible. Nonetheless, we are moving forward precision medicine and targeted therapies, as already done in other fields, such as in oncology. Therefore, all efforts to better understand such a complex disease as PD should be considered as starting points toward new targeted approaches. Then, the aim would be to smooth and simplify them, to make them accessible.

Therefore, in this prospective study we measured motor performances of a cohort of PD patients from the earliest stage of the disease, before the beginning of a dopamine replacement therapy, up to 30 months after the diagnosis. Our results highlight the possibility of technology-based deep motor phenotyping and its significant role in defining innovative but reliable prognostic and therapy-response biomarkers.

## Methods

### Patients

Forty patients diagnosed with PD according to the MDS clinical diagnostic criteria^33^ were consecutively enrolled at their first visit at “Tor Vergata” movement disorders outpatient clinic. They were asked to come with a maximum latency of 3 weeks from enrollment for the first clinical and kinematic evaluation (T0 time).

The study was conducted in agreement with ethical principles of Helsinki declaration. Informed consent was obtained from all participants after receiving full explanation of the procedures approved by the ethical Committee of the hospital (RS 34/17).

### Ratings

The clinical evaluations consisted of a general neurological assessment, record of dopaminergic therapy with calculation of the Levodopa Equivalent Daily Dose (LEDD) and quantification of motor and non-motor symptoms by means of the MDS-Unified Parkinson’s Disease Rating Scale part III (MDS-UPDRS III), and the Non-Motor Symptoms Scale (NMSS). In addition, we performed rest, tremor and motor impairment evaluations from measurements gathered by means of wearable electronic sensors (wearables, hereafter).

### Wearables

**Fig. 2:**
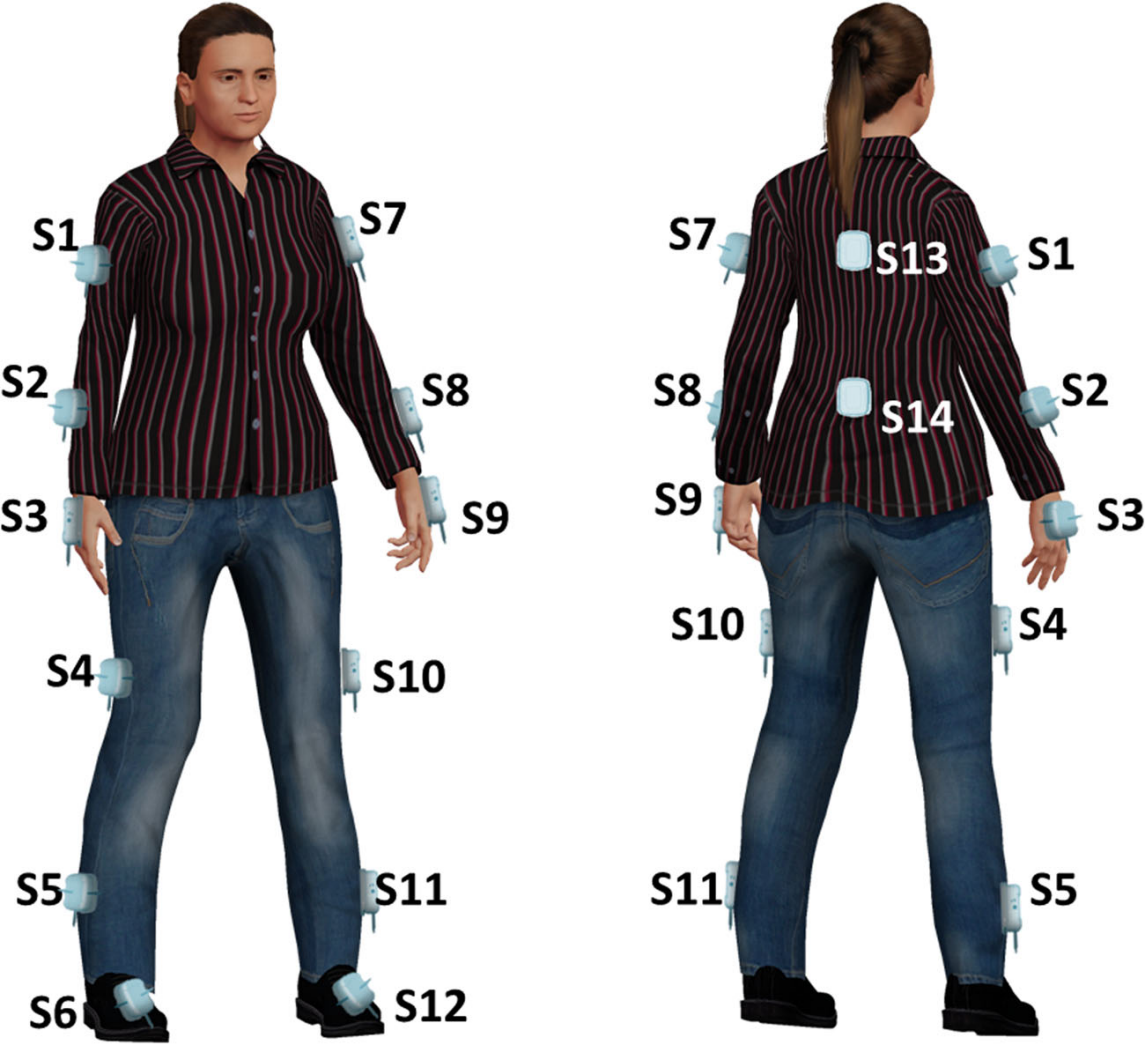
Position of wearable sensors. For RT, PosT, and RAHM tasks S3 and S9 were analyzed. For LA S4 and S10, for TT S6 and S12. For PT we used S5, S11 ans S13. For TUG S1, S2, S4, S5, S7, S8, S10, S11, S13, and S14.

Wearables consisted of validated Movit G1 devices (by Captiks, Rome, Italy)^13,14,22,38^ with three-axis accelerometer and three-axis gyroscope sensors on-board. The Movit G1 were placed on body segments, by means of Velcro™ strips, in particular on the body back, on each forearm and arm, on each upper and lower leg, on the dorsum of each hand and of each foot, for a total of fourteen sensors (Fig. 2)^20,22^. Each Movit G1 gathered and synchronously sent data to a receiver connected to a personal computer running the Motion Studio application (by Captiks, Rome, Italy) for data tracking and recording.

### Motor tasks and features

According to UPDRS part III, patients performed seven motor tasks, in particular:

- Rapid Alternating Hand Movements (RAHM, MDS-UPDRS item 3.6).
- Leg Agility (LA, MDS-UPDRS item 3.8).
- Toe Tapping (TT, MDS-UPDRS item 3.7).
- Timed-Up-and-Go (TUG, grossly MDS-UPDRS items 3.9 plus 3.10), i.e., rising from a chair, walking for 6 meters, turning 180 degrees, walking back and sitting.
- Pull Test (PT, MDS-UPDRS item 3.12).
- Postural Tremor and Rest Tremor of the upper limbs (PosT and RT, grossly MDS-UPDRS item 3.15 and 3.17).

The kinematic features extracted from each motor task, previously defined^20,22^, are described in Table 5.

**Table 5:** Kinematic features’ description.

Feature (task)	Description
Power (PosT and RT)	Power at the frequency range 3–12 Hz of hand tremor.
Amplitude (PosT and RT)	Amplitude at the fundamental frequency (f0) of hand tremor.
Amp (RAHM, LA and TT)	Normalized average peaks’ amplitude of the angular velocity.
CV (RAHM, LA and TT)	Coefficient of variation (CV) between consecutive peaks of the angular velocity. It measures the rhythm.
Amp_CV (RAHM, LA and TT)	Coefficient of variation of the peaks’ amplitude. It measures the regularity of the movement.
Asym (RAHM, LA and TT)	Difference in peaks’ amplitude between the faster and slower limb divided by the larger value. It measures the asymmetry.
Amp_Decr (RAHM, LA and TT)	Ratio of the average angular rate in the first third of oscillation to the last third. It measures the amplitude reduction over time.
Power (RAHM, LA and TT)	Maximum amplitude of the power spectral density of the angular velocity.
Asym_Power (RAHM, LA and TT)	Difference of power spectral density’s peaks between the faster and slower limb divided by the larger value.
ROM (PT)	Range of motion of the trunk movements in the three directions after the push (three features in total).
Number of Steps (PT)	Number of strides after the subject has been pushed.
Jerk (PT)	Derivative of the acceleration in transverse and sagittal direction.
Area (PT)	Ellipse area that comprises 95% of the values of the accelerations in transverse and sagittal directions around their mean values.
Range (PT)	Range of acceleration and angular velocity data in 3D (6 features in total).
Duration metrics (TUG)	Comprise TUG Time (duration of TUG test), Sit-To-Walk Time (from the beginning of standing to the beginning of walking), Walk Time (duration of the walking), Turning Time (duration of 180° turn), and Turn-To-Sit Time (time required to turn and sit on the chair).
Number of Steps (TUG)	Number of strides.
Gait metrics (TUG)	Comprise the duration of Stance, Swing and Double Support, and their CVs.
Cadence (TUG)	Number of steps per minute.
ROM Trunk (TUG)	Range of motion of the trunk movements while walking (three features in total).
Flex Arm, Flex Leg (TUG)	Flexion of arms and legs in the sagittal plane. This parameter is computed using the more affected side.
Asym Arm, Asym Leg (TUG)	Difference in the angular flexion values between the faster and slower arm/leg divided by the larger value.
Average Vel (TUG)	Mean angular velocity of arms, forearms, and thighs during walking.
Rms Turning (TUG)	Root mean square acceleration values of the trunk during the turning phase (three features in total).
Turning Vel (TUG)	180 °divided by the duration of turning (°/s)
Peak Turning Vel (TUG)	Maximum angular velocity of trunk during the turning phase.
Steps Turning (TUG)	Number of strides during the turning phase.
Rms Sitwalk (TUG)	Root mean square acceleration values of the trunk while standing up (three features in total).
Range Trunk Sitwalk (TUG)	Range of acceleration along the sagittal axis of the trunk while standing up.
Peak Vel Sitwalk (TUG)	Maximum angular velocity of trunk in in the sagittal plane while standing up.
Average Vel Sitwalk (TUG)	Mean angular velocity of trunk in in the sagittal plane while standing up.
Flex Trunk Sitwalk (TUG)	Flexion of the trunk in the sagittal plane.

### Work timeline

**Fig. 3:**
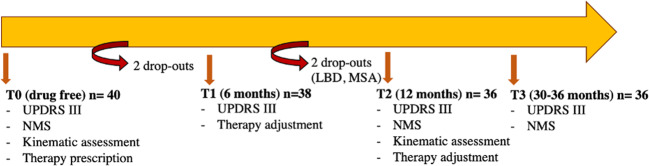
Work timeline of the clinical and kinematic assessments and interventions. Study design: clinical and kinematic assessments and interventions for each time point.

Figure 3 shows the work timeline.

At time T0, we clinically assessed 40 de novo drug-free PD patients by means of MDS-UPDRS III, NMSS rating scales, and measured their rest and motor impairments by means of wearables. For each patient we prescribed a therapy according to the clinical status.

At T1, 6 months later, a standard clinical follow-up visit was provided. We clinically reevaluated patients in order to adjust the dopaminergic therapy as needed. Two patients leaved the study on their own initiative.

At T2, 12 months later, we readopted MDS-UPDRS III, NMSS, wearables, and readjusted the dopaminergic therapy as necessary. We excluded two patients from the study because one diagnosed with multiple system atrophy and the other with Lewy body dementia.

At T3, within 30–36 months later, we performed further clinical evaluations by means of MDS-UPDRS III and NMSS.

### Statistical analysis

The analysis of variance (ANOVA) was carried out on the kinematic and clinical variables to evaluate the efficacy of Levodopa therapy and to identify technology-based therapy-response biomarkers. In particular, ANOVA model for repeated measures was performed with time (baseline, 6 months, and 12 months’ follow-up) as within-subjects factor. A *p* value <0.05 was considered significant.

The standardized response mean (SRM) was used to assess the responsiveness of each variable to the therapy. The SRM is calculated as TT/SDC, where TT is the mean change between T2 and T0 and SDC is the standard deviation of the change. Empirically, an SRM value of 0.20 represents a small, 0.50 a moderate, and 0.80 a large responsiveness, respectively.

In order to identify possible early prognostic technology-based biomarkers, a Pearson correlation test was performed between the UPDRS III score variations from T0 to T3 and baseline kinematic features of all patients. Then, a multiple linear regression model was made to predict the long-term motor outcome using T0 kinematic measures. In order to take into account the different amount of motor impairment at T0 among patients, and different MDS-UPDRS III scores, we used of the percentage of variation rather than the raw score variation to evaluate patients’ motor progression. We therefore considered the percentage of variation (%) in UPDRS between T3 and T0 as the dependent variable. The independent variables were selected among the features that showed a better correlation with the UPDRS III change and showed an association at univariate analysis (*p* < 0.10), as appropriate. LEDD values at T3 were included in the model as independent variable to test for any influence in the outcome related to the different dopaminergic drugs dosage among patients. The model was tested for multicollinearity, examination of residual distribution, and heteroscedasticity.

### Reporting summary

Further information on research design is available in the Nature Research Reporting Summary linked to this article.

## Supplementary information

Reporting Summary

## Data Availability

The data collected during this study are available from the corresponding author upon reasonable request from qualified individuals.

## Code availability

The codes used in this study are available from the corresponding author upon reasonable request from qualified individuals.

## References

[1] Ray Dorsey E, George BP, Leff B, Willis AW (2013). The coming crisis: obtaining care for the growing burden of neurodegenerative conditions. Neurology..

[2] Ascherio A, Schwarzschild MA (2016). The epidemiology of Parkinson’ s disease: risk factors and prevention. Lancet Neurol..

[3] Parnetti L (2019). CSF and blood biomarkers for Parkinson’s disease.. Lancet Neurol..

[4] Lotankar S, Prabhavalkar KS, Bhatt LK (2017). Biomarkers for Parkinson’s disease: recent advancement.. Neurosci. Bull..

[5] Fereshtehnejad S-M, Zeighami Y, Dagher A, Postuma RB (2017). Clinical criteria for subtyping Parkinson’s disease: biomarkers and longitudinal progression. Brain.

[6] Simuni T (2018). Longitudinal change of clinical and biological measures in early Parkinson’s disease: Parkinson’s progression markers initiative cohort. Mov. Disord..

[7] Rocha, A. P. et al. Parkinson’s disease assessment based on gait analysis using an innovative RGB-D camera system. In *2014 36th Annual International Conference of the IEEE Engineering in Medicine and Biology Society*. 3126–3129 (EMBC*,* 2014). 10.1109/EMBC.2014.6944285 25570653

[8] Ťupa O (2015). Motion tracking and gait feature estimation for recognising Parkinson’s disease using MS Kinect.. Biomed. Eng..

[9] Breit S, Spieker S, Schulz JB, Gasser T (2008). Long-term EMG recordings differentiate between parkinsonian and essential tremor. J. Neurol..

[10] Argaud S (2016). Does facial amimia impact the recognition of facial emotions? An EMG study in Parkinson’s disease. PLoS ONE.

[11] Rissanen S (2007). Analysis of surface EMG signal morphology in Parkinson’s disease. Physiol. Meas..

[12] Patel S (2010). A web-based system for home monitoring of patients with Parkinson’s disease using wearable sensors. IEEE Trans. Biomed. Eng..

[13] Ricci M (2019). Wearable-based electronics to objectively support diagnosis of motor impairments in school-aged children. J. Biomech..

[14] Alessandrini M (2017). Body-worn triaxial accelerometer coherence and reliability related to static posturography in unilateral vestibular failure. Acta Otorhinolaryngol. Ital..

[15] Kim JW (2011). Quantification of bradykinesia during clinical finger taps using a gyrosensor in patients with Parkinson’s disease. Med Biol. Eng. Comput.

[16] Giuberti M (2015). Automatic UPDRS evaluation in the sit-to-stand task of parkinsonians: Kinematic analysis and comparative outlook on the leg agility task. IEEE J. Biomed. Heal Inform..

[17] Saggio, G. Are sensors and data processing paving the way to completely non-invasive and not-painful medical tests for widespread screening and diagnosis purposes? in *BIODEVICES 2020—13th International Conference on Biomedical Electronics and Devices, Proceedings; Part of 13th International Joint Conference on Biomedical Engineering Systems and Technologies, BIOSTEC 2020*. 207–214, (SciTePress, 2020).

[18] Espay AJ (2017). Technology in Parkinson disease: challenges and opportunities. Mov. Disord..

[19] Rovini E, Maremmani C, Cavallo F (2017). How wearable sensors can support parkinson’s disease diagnosis and treatment: a systematic review.. Front. Neurosci..

[20] Di Lazzaro G (2019). Technology-based objective measures detect subclinical axial signs in untreated, de novo Parkinson’s disease. J. Parkinsons Dis..

[21] Ricci M (2019). Wearable electronics assess the effectiveness of transcranial direct current stimulation on balance and gait in Parkinson’s disease patients. Sensors.

[22] Ricci M (2019). Assessment of motor impairments in early untreated Parkinson’s disease patients: the wearable electronics impact.. IEEE J. Biomed. Heal. Inform.

[23] Espay AJ (2019). A roadmap for implementation of patient-centered digital outcome measures in Parkinson’s disease obtained using mobile health technologies. Mov. Disord..

[24] Artusi CA (2018). Integration of technology-based outcome measures in clinical trials of Parkinson and other neurodegenerative diseases. Park Relat. Disord..

[25] Jankovic J, Watts RL, Martin W, Boroojerdi B (2007). Transdermal rotigotine: double-blind, placebo-controlled trial in Parkinson disease. Arch Neurol..

[26] Schapira AHV (2017). Assessment of safety and efficacy of safinamide as a levodopa adjunct in patients with Parkinson disease and motor fluctuations a randomized clinical trial.. JAMA Neurol..

[27] Ferreira JJ (2016). Opicapone as an adjunct to levodopa in patients with Parkinson’s disease and end-of-dose motor fluctuations: a randomised, double-blind, controlled trial. Lancet Neurol..

[28] Bond AE (2017). Safety and efficacy of focused ultrasound thalamotomy for patients with medication-refractory, tremor-dominant Parkinson disease a randomized Clinical trial. JAMA Neurol..

[29] Bologna M (2016). Bradykinesia in early and advanced Parkinson’s disease. J. Neurol. Sci..

[30] Espay AJ (2011). Differential response of speed, amplitude, and rhythm to dopaminergic medications in Parkinson’s disease. Mov. Disord..

[31] Ling H, Massey LA, Lees AJ, Brown P, Day BL (2012). Hypokinesia without decrement distinguishes progressive supranuclear palsy from Parkinson’s disease. Brain.

[32] Lipsmeier F (2018). Evaluation of smartphone-based testing to generate exploratory outcome measures in a phase 1 Parkinson’s disease clinical trial. Mov. Disord..

[33] Postuma RB (2015). MDS clinical diagnostic criteria for Parkinson’s disease. Mov. Disord..

[34] Verghese J (2014). Motoric cognitive risk syndrome multicountry prevalence and dementia risk. Neurology.

[35] Cohen, J. A., Verghese, J. & Zwerling, J. L. Cognition and gait in older people. **93**, 73–77 (Elsevier Ltd, 2016). 10.1016/j.maturitas.2016.05.005 27240713

[36] Yoo JE (2020). Timed up and go test and the risk of Parkinson’s disease: a nation-wide retrospective cohort study. Mov. Disord..

[37] Markello RD (2021). Multimodal phenotypic axes of Parkinson’s disease. npj Park. Dis..

[38] Costantini G (2018). Towards the enhancement of body standing balance recovery by means of a wireless audio-biofeedback system. Med. Eng. Phys..

